# Versatile whey acidic protein four-disulfide core domain proteins: biology and role in diseases

**DOI:** 10.3389/fcell.2024.1459129

**Published:** 2024-09-04

**Authors:** Yifan Wen, Nan Jiang, Zhen Wang, Yuanyuan Xiao

**Affiliations:** ^1^ Department of Medical Genetics, West China Second University Hospital, Sichuan University, Chengdu, Sichuan, China; ^2^ Key Laboratory of Birth Defects and Related Diseases of Women and Children, Ministry of Education, Sichuan University, Chengdu, China; ^3^ Division of Liver Surgery, Department of General Surgery and Laboratory of Liver Surgery, State Key Laboratory of Biotherapy, West China Hospital, Sichuan University, Chengdu, China

**Keywords:** WFDC proteins, cancer, inflammatory disease, biomarker, signaling/signaling pathways, heart failure

## Abstract

The Whey acidic protein four-disulfide core (WFDC) protein family consists of proteins with one or more WFDC domains which are ubiquitously expressed throughout the body of human and perform a wide range of functions, including antiprotease, antibacterial, and immunomodulatory functions. Aberrant expression of WFDC proteins is associated with human diseases. However, review on the WFDC protein family is limited and insufficient. Furthermore, a systematic summary of the underlying mechanisms of WFDC protein activity is lacking. In this review, we give a summary of the structural basis and molecular function of these proteins and review the immune regulatory mechanisms and signaling pathways of WFDC proteins in the development of certain diseases. Furthermore, we discuss the diagnostic and prognostic potential of multiple WFDC proteins in the aforementioned conditions, as well as their prospective use. At last, we also discuss the progress of WFDC protein in clinical trials and put forward some research difficulties and the directions of follow-up research. Our review highlights the functional diversity and clinical significance of WFDC proteins family, while providing potential targets for drug development and innovative therapeutic strategies, this review lays the foundation and direction for future research on WFDC proteins.

## 1 Introduction

**FIGURE 1:**
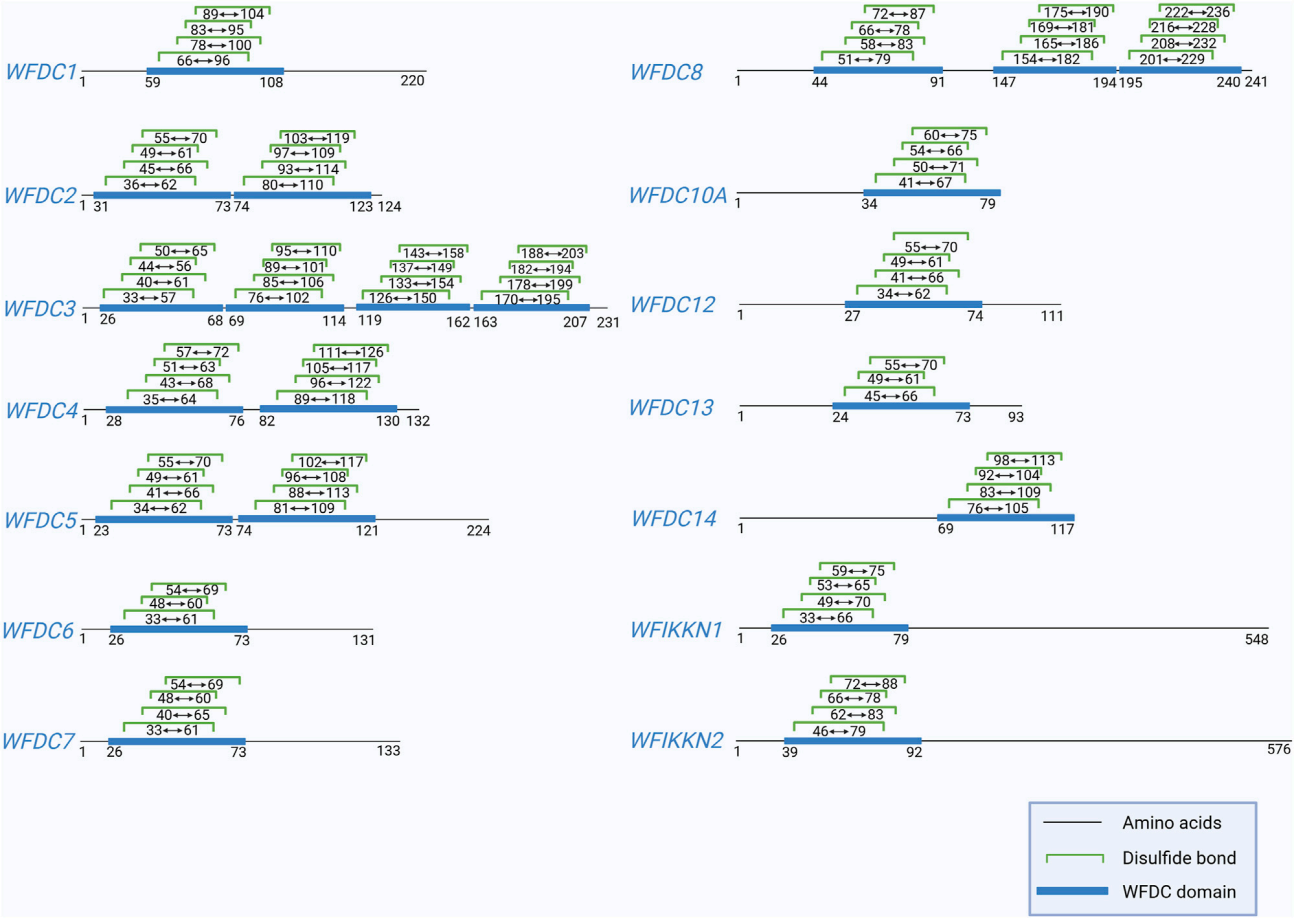
This figure shows the positions of the disulfide bonds in WFDC domain of human WFDC proteins, except for WFDC9, WFDC10B, WFDC11, and ANOS1, due to insufficient data. And the information is obtained from UniProt. Created with BioRender.com.

The Whey acidic protein four-disulfide core (WFDC) proteins are characterized by a sequence comprising one or more WFDC domains—a sequence of 40–50 amino acids, including eight conserved cysteine residues, that form 4 disulfide bonds (Ranganathan et al., 1999) (Figure 1). The WFDC domain structure includes a central β-fold with two outer segments linked by a loop that connects the protease binding sites (Francart et al., 1997). The term “four-disulfide core” was originally used to describe the structure of four distinct intramolecular disulfide bonds (Drenth et al., 1980). This motif appears in various unrelated proteins, including wheat germ agglutinin, several snake venom neurotoxins, and whey acidic protein (WAP), which is the principal whey protein in mouse milk (Piletz et al., 1981). Mouse WAP was later recognized as the prototype of this WFDC domain subfamily. Consequently, proteins with similar disulfide bond arrangements are now referred to as whey acidic protein four-disulfide core proteins, although the term WAP is still commonly used. To date, 18 WFDC domain-containing proteins have been identified in humans (Bingle and Vyakarnam, 2008). Other mammals with WFDC domain proteins include camel (cWAP), rabbit (rabWAP), rat (ratWAP) and pig (pWAP) (Ranganathan et al., 1999). Following the discovery of WFDC domain-containing proteins in vertebrates, homologs were first found in invertebrates, such as shore crabs. For instance, antimicrobial peptides from crustaceans also contain the WFDC domain in their carboxy-terminal region, highlighting its role as a conserved scaffold used by nature to produce functionally diverse proteins over millions of years (Smith et al., 2008).

The WFDC gene cluster, spanning over 700 kb on human chromosome 20, is divided into two subloci: the centromeric and the telomeric regions. The cluster contains 14 of the 18 WFDC genes (Hurle et al., 2007). Specifically, the centromeric sublocus contains the genes WFDC5, WFDC12, WFDC14, and WFDC4, while the telomeric sublocus includes WFDC2, WFDC6, WFDC7, WFDC8, WFDC9, WFDC10A, WFDC11, WFDC10B, WFDC13, and WFDC3 (Ferreira et al., 2013; Clauss et al., 2002). The majority of these genes encode small secretory proteins. A subset of WFDC genes located on chromosome 20q, consisting of WFDC8, WFDC7, and WFDC6, contains both Kunitz and WAP domain, classifying them as protease inhibitors of the WAP/Kunitz type (Clauss et al., 2002). The ANOS1, WFIKKN1, WFIKKN2, and WFDC1 genes, located outside the WFDC cluster, they all encode larger multi-domain proteins, except for WFDC1, which contains only a WFDC domain (Larsen et al., 1998). The 14 genes within the WFDC cluster show signs of adaptive evolution, suggesting their association with reproduction and immunity (Ferreira et al., 2013). Especially at closer evolutionary distances, this genomic locus has been reported to be one of the most rapidly divergent regions among the genomes of humans and chimpanzees (Hurle et al., 2007). In contrast, the four WFDC genes outside the cluster are more randomly distributed and exhibit higher conservation in the human gene family, underscoring the significance of researching WFDC genes across different organisms.

WFDC proteins are expressed in various organs in healthy individuals (Table 1). Notably, WFDC14 is primarily expressed in the epithelium and inflammatory cells (Moreau et al., 2008; Williams et al., 2006), WFDC7 is mainly found in the epididymis and testes (Yenugu et al., 2004), WFDC12 is presented in the prostate, skin, lungs, and esophagus (Clauss et al., 2005), while WFDC4 and WFDC2 are nearly ubiquitous (Wang et al., 1999; Bingle et al., 2006; McNeely et al., 1997; Sallenave et al., 1994). However, the expression of WFDC genes is primarily observed in the reproductive and respiratory organs (Bingle and Vyakarnam, 2008). Additionally, WFDC gene expression is modulated by cytokines and may be aberrantly expressed in tumor cells (Table 1). For example, WFDC1 is highly expressed in prostate cancer (Larsen et al., 1998; Larsen et al., 2000), and WFDC2 shows abnormal expression in various tumor cells (Wang et al., 1999; Bingle et al., 2006). These WFDC proteins have demonstrated significant potential in applications, due to their diverse functions, including antiprotease, antibacterial, antiviral, and immunomodulatory activities (Williams et al., 2006; Scott et al., 2011). Furthermore, they are associated with the onset, diagnosis, and treatment of various clinical diseases. While WFDC proteins are primarily known for their antiprotease functions, such as inhibiting cathepsin G, their protease inhibition capabilities have broad biological and therapeutic implications, emphasizing the significance of the WFDC protein family.

**TABLE 1:** The expression and related mechanism of WFDC proteins.

WFDC protein	Alternative names	Sites of expression in human	Abnormal expression of diseases	Related mechanisms	Type of trials	References
WFDC1	PS20	Prostate	Downregulated in prostate cancer and upregulated in preeclampsia	In prostate cancer, WFDC1 inhibits the expansion of CD8^+^ T cells and NK cells and regulate the COX-2 expression to inhibit epithelial growth	*Invitro* studies in cell lines and patient	Rajakumar et al. (2011), Hickman et al. (2016a), Hickman et al. (2012)
WFDC2	HE4, WAP5	Ubiquitous	Upregulated in ovarian cancer, lung cancer, interstitial disease, and heart failure, downregulated in prostate cancer	In ovarian cancer, overexpressed WFDC2 is associated with the proliferation, metastasis, and invasion of it, attributed to the change of PI3K/AKT and JAK/STAT3 signaling pathways In prostate cancer, WFDC2 inhibits prostate cancer metastasis by inhibiting the activation of EGFR	*Invitro* studies in cell lines	Clauss et al. (2005), Wang et al. (2015), Gao et al. (2011), Xiong et al. (2020), Piek et al. (2017), Nagy et al. (2014), Zhang et al. (2020), James et al. (2018), Lou et al. (2019)
WFDC3	WAP14	Not determined	Not determined	Not determined	Not determined	—
WFDC4	SLPI, ALP, WAP4	Ubiquitous	upregulated in ovarian cancer and psoriasis	In ovarian cancer, WFDC4 affects the PI3-AKT signaling pathway and stimulates ovarian cancer invasion by weakening MMP-9 release In psoriasis, WFDC4 stimulates pDCs to secret IFN-α by activating the extracellular DNA	*Invitro* studies in cell lines and preclinical studies in mouse models	Clauss et al. (2005), Sun et al. (2022), Hoskins et al. (2011), Zabieglo et al. (2015), Skrzeczynska-Moncznik et al. (2012), Wang et al. (2019)
WFDC5	WAP1	Skin	Not determined	Not determined	Not determined	Kalinina et al. (2020)
WFDC6	WAP6	Not determined	Not determined	Not determined	Not determined	—
WFDC7	Eppin, WAP7, SPINLW1	Testes, epididymis	Not determined	Not determined	Not determined	Clauss et al. (2005), O’Rand et al. (2006)
WFDC8	WAP8	Not determined	Not determined	Not determined	Not determined	—
WFDC9	WAP9	Not determined	Not determined	Not determined	Not determined	—
WFDC10A	WAP10	Not determined	Not determined	Not determined	Not determined	—
WFDC10B	WAP12	Not determined	Not determined	Not determined	Not determined	—
WFDC11	WAP11	Not determined	Not determined	Not determined	Not determined	—
WFDC12	WAP2	Prostate, skin, lung	Upregulated in psoriasis and AD	In psoriasis, WFDC12 affects the activation of the retinoic acid signaling pathway and regulates the infiltration of DC cells in the skin lesions and lymph nodes, inducing Th1 cell differentiation and increasing the secretion of IFN-γ In AD, the overexpressed-WFDC12 may affect the development of AD by promoting ALOX12/15 metabolism and PAF accumulation	Preclinical studies in mouse models	Glasgow et al. (2015), Zhao et al. (2022), Li et al. (2023)
WFDC13	WAP13	Not determined	Not determined	Not determined	Not determined	—
WFDC14	PI3, elafin, WAP3	Epithelium/Inflammatory cells	Upregulated in psoriasis	WFDC14 inhibits LPS-induced phosphorylation of JNK and subsequent phosphorylation of AP-1 subunit ATF2 and c-Jun	*Invitro* studies in cell lines	Clauss et al. (2005), Butler et al. (2006)
ANOS1	KAL-1	Not determined	Not determined	Not determined	Not determined	—
WFIKKN1	WFIKKN	Pancreas, thymus, liver	Not determined	Not determined	Not determined	Trexler et al. (2001)
WFIKKN2	WFIKKNRP	Ovary, testes, brain	Not determined	Not determined	Not determined	Trexler et al. (2002)

The expression of WFDC proteins in human and different diseases, related mechanisms in diseases and the type of trials. PS20, prostate stromal 20; NK, cell, natural killer cell; COX-2, cyclooxygenase-2; HE4, human epididymis protein 4; SLPI, secretory leukocyte protease inhibitor; pDC, plasmacytoid dendritic cell; AD, atopic dermatitis; Th1 cell, T-helper 1 cell; PI3, peptidase inhibitor 3; LPS, lipopolysaccharide.

The preceding discussion highlights the WFDC protein family’s broad functional spectrum and significant potential, making it valuable for drug development and disease treatment based on its diverse functions. Furthermore, aberrant expression of WFDC proteins is linked to the pathogenesis of various diseases and cancers, offering novel insights for diagnosis and treatment strategies. The necessity and significance of studying WFDC proteins are evident, underscoring the value of this review.

## 2 The biology of the WFDC proteins

The WFDC protein family is characterized by a distinctive structural domain known as the WFDC domain, which is integral to their diverse biological functions. These proteins are notable for their antiprotease activities as well as their diverse roles in host defense, including antibacterial, antiviral, and immunomodulatory functions. Consequently, the WFDC proteins are implicated in a range of physiological processes and disease states, from inflammation and infection to immune system regulation. This section will explore the various functional aspects of WFDC proteins, focusing on their antiprotease activity, antibacterial properties, antiviral effects, and immunomodulatory roles.

### 2.1 Antiprotease function

**FIGURE 2:**
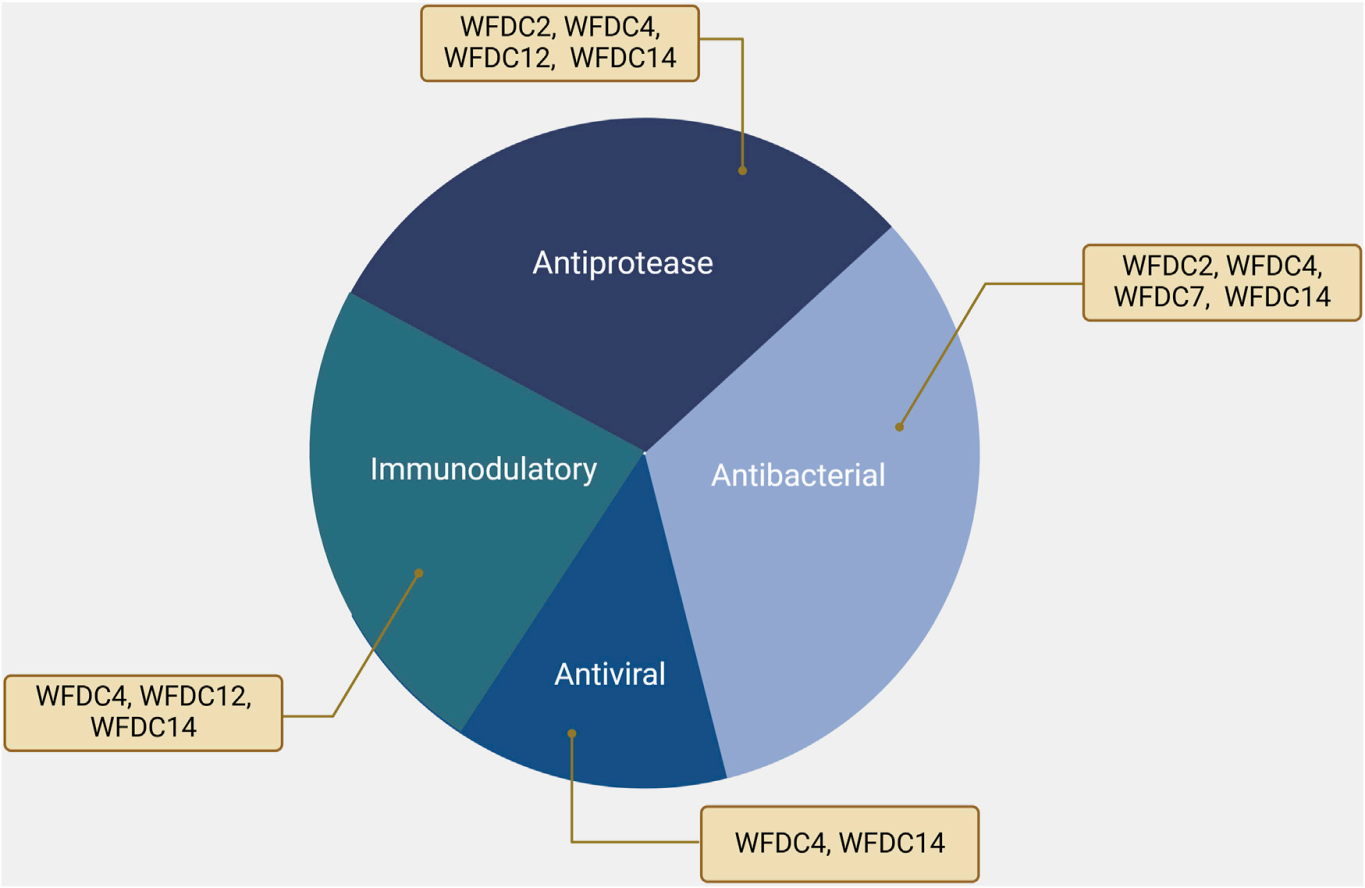
The functions of WFDC proteins, including WFDC4, WFDC14, WFDC12 and WFDC2. Among them, WFDC4 and WFDC14 have antiprotease, antibacterial, antiviral, and immunomodulatory functions. For WFDC12, it shows the antiprotease and immunomodulatory activity. And WFDC2 can inhibit several proteases and bacteria. Created with BioRender.com.

The serine protease inhibitory activity of WFDC proteins is a well-supported aspect of their function (Hurle et al., 2007). Many WFDC family members exhibit antiprotease activity, which is attributed to the structure of the WFDC domains (Bingle and Vyakarnam, 2008). Specifically, proteins such as WFDC4, WFDC14, WFDC12, and WFDC2, have been shown to exhibit antiprotease activity, as depicted in Figure 2 (Moreau et al., 2008; Williams et al., 2006; Glasgow et al., 2015; Chhikara et al., 2012).

WFDC4, one of the most extensively studied bidomain WFDC proteins, inhibits various proteases, including neutrophil elastase (NE), trypsin, chymotrypsin, and cathepsin G, but does not affect proteinase 3 (Drenth et al., 1980). The inhibitory sites for elastase and chymotrypsin are leucine 72 in the C-terminal domain, whereas trypsin inhibitors typically have lysine or arginine as the inhibitory site (Eisenberg et al., 1990). WFDC4 is abundantly found in diverse secretions such as bronchial, nasal, seminal, and cervical mucus (Bingle and Vyakarnam, 2008), playing a crucial role in the antiprotease defense of these tissues. This suggests potential therapeutic applications for WFDC4 in treating diseases associated with protease-related tissue damage and inflammation.

WFDC14 inhibits neutrophil elastase and suppresses proteinase 3, unlike WFDC4, which does not inhibit proteinase 3. (Sallenave, 2010). At the same time, recombinant WFDC12 (rWFDC12) exhibits a dose-dependent inhibitory effect on cathepsin G, although its suppression of elastase and proteinase 3 is less pronounced (Glasgow et al., 2015). While WFDC2 can inhibit a variety of proteases, including trypsin, prostate-specific antigen (PSA), proteinase K, pepsin, papain, etc., showing cross-class protease inhibition (Chhikara et al., 2012).

This section discusses the impact of the WFDC domain on the antiprotease function of this protein family. Studies have shown that the antiprotease activity of WFDC4 is specific to the C-terminal WFDC domain (Eisenberg et al., 1990), while WFDC14, which contains only a WFDC domain, also exhibits antiprotease activity. Furthermore, the spacing between cysteine residues in the WFDC domain is critical for their antiprotease function (Eisenberg et al., 1990; Kramps et al., 1990). And in the C-terminal WFDC domain of WFDC4, the interval between conserved residues is consistent (Bingle and Vyakarnam, 2008). Researchers have also observed this consistent spacing in antiprotease family members across different species. (Ranganathan et al., 1999). Comparison of all human WFDC domains reveals that the specific spacing between cysteine residues 1, 2, and, 3 (respectively, 6 and 8 amino acids) is present only in the C-terminal WFDC domain of WFDC4 and the WFDC domain of WFDC14. However, in the other WFDC domains of humans, the interval between cysteine 2 and 3 ranges in three, four, or seven residues, which may result in insufficient space for the WFDC domain to generate protease inhibition sites during the folding process (Bingle and Vyakarnam, 2008). WFDC domains with varying cysteine spacing exhibit reduced or inconsistent antiprotease activity, highlighting the importance of specific structural configurations for functional efficacy. This structural variation underscores the importance of precise domain architecture in determining the biological roles of WFDC proteins.

### 2.2 Antibacterial function

Beyond their antiprotease functions, WFDC proteins have broader biological significance. Members such as WFDC4, WFDC14, WFDC7, and WFDC2 exhibit a spectrum of additional roles in host defense mechanisms (Figure 2). Apart from their antiprotease activity, these proteins have been identified to possess significant antibacterial properties, combating a variety of pathogens.

The antimicrobial activity of WFDC4 and WFDC14 is hypothesized to be accomplished by their cationic charge (+12 and +3, respectively), which disruption of bacterial cell membranes independently of their antiprotease function (Williams et al., 2006; Baranger et al., 2008). Both WFDC4 and WFDC14 exhibit inhibitory activity against Gram-positive and Gram-negative bacteria (Baranger et al., 2008; Hiemstra et al., 1996). WFDC4 is effective against *Pseudomonas aeruginosa* and *Staphylococcus aureus*, common pathogens in upper respiratory infections (Williams et al., 2006; Hiemstra et al., 1996). And for WFDC4, its N-terminal domain exhibits stronger antibacterial activity than the C-terminal domain, however, the synergistic effect of both domains resulting in the strongest antimicrobial activity (Hiemstra et al., 1996). WFDC14 can inhibit *P*. *aeruginosa* and *S*. *aureus* (Meyer-Hoffert et al., 2003), and it also exhibits antifungal activity against *Aspergillus fumigatus* and *Candida albicans*, both of which are famillar pathogenic fung (Baranger et al., 2008). These bacterial or fungal infections can cause tissue damage and inflammation and the antimicrobial properties of WFDC4 and WFDC14 suggest their potential use in treating such diseases.

WFDC7, primarily expressed in the testicles and epididymis, coats ejaculated sperm, which is bound and saturated by semenogelin, providing antibacterial activity that aids in sperm agglutination (O’Rand et al., 2006). As mentioned earlier, WFDC2 inhibits proteinase K, a member of the subtilisin-like protease family. Additionally, this protease family often act as the virulence factor in many pathogenic fungi (Siezen et al., 1991; Stleger, 1995; Huang et al., 2004), suggesting that WFDC2 may offer protection at its expression site.

WFDC proteins from non-human species also exhibit protease resistance. Crustins, a family of antimicrobial peptides found in Pancrustacea, are another example of WFDC domain-containing proteins with antibacterial activity (Smith et al., 2008). These peptides, characterized by their WFDC domains, combat various bacterial pathogens, including Gram-positive but not Gram-negative bacteria (Smith et al., 2008). SWAM1 and SWAM2, proteins described in mice, also exhibit significant antibacterial activities (Hagiwara et al., 2003). Similarly, omwaprins, isolated from snake venom, demonstrate potent antibacterial effects (Nair et al., 2007), highlighting the diverse range of WFDC domain-containing proteins with antimicrobial properties.

### 2.3 Antiviral function

**FIGURE 3:**
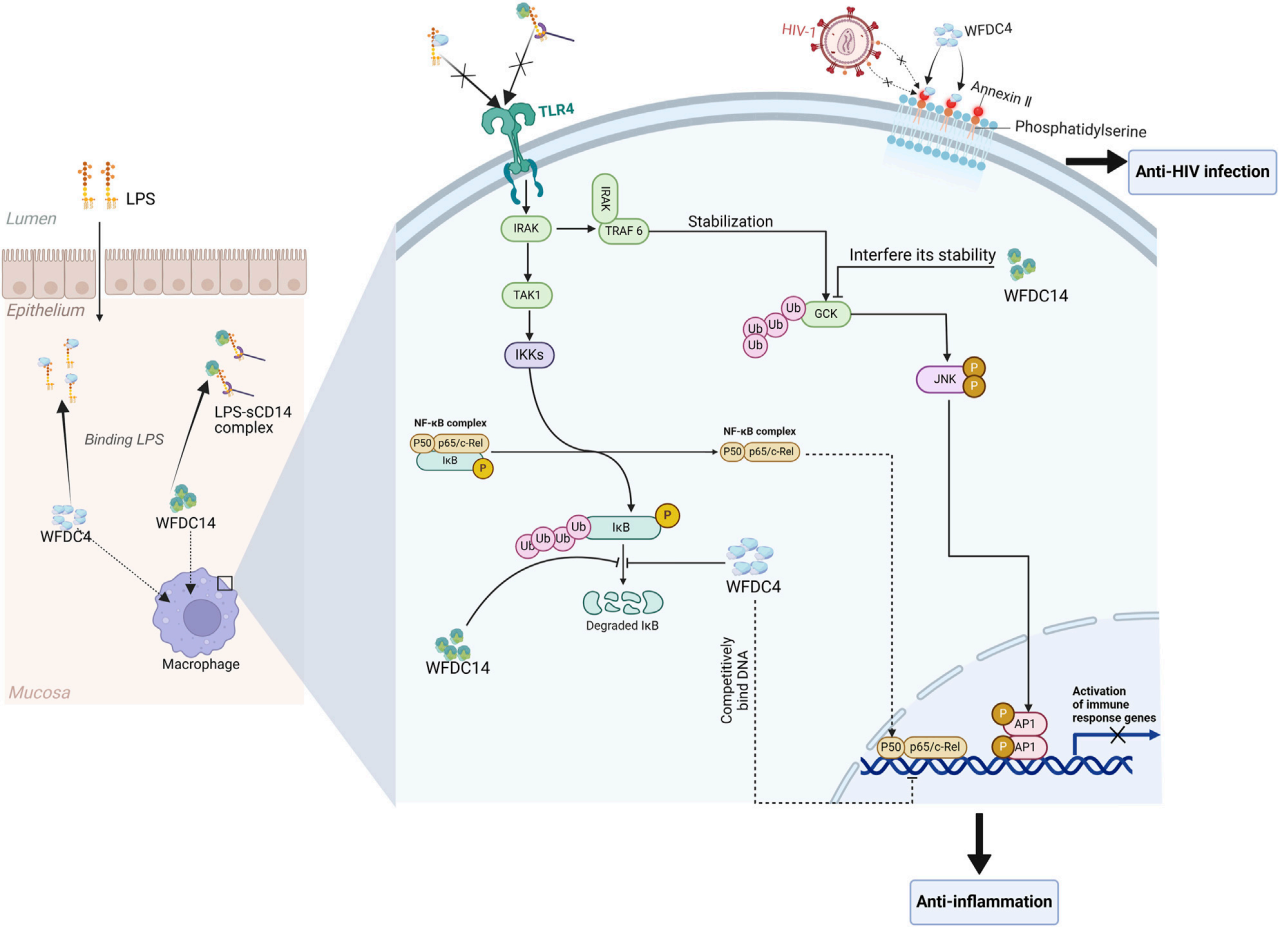
The biological functions of WFDC proteins are involved in several molecular mechanisms. For the anti-HIV ability, WFDC4 can bind to annexin Ⅱ, which is a cofactor for the HIV-1 infection. And in the immunomodulatory function, WFDC4 and WFDC14 can inhibit the responses mediated by LPS. Extracellularly, WFDC4 can neutralize the LPS as well as WFDC14. And intracellularly, the degradation of IκBα which will activate the NF-κB pathway can be inhibited by WFDC4 and WFDC14. Moreover, WFDC4 can competitively bind to NF-κB DNA sites at the gene promoter regions to inhibit the related gene expression. TLR4, toll-like receptor 4. GCK, germinal center kinase. Created with BioRender.com.

Some studies suggest that the WFDC4 and WFDC14 may possess antiviral properties. WFDC4 has been found to have the ability to inhibit HIV-1 infection of macrophages (McNeely et al., 1995) and can competitively bind to annexin II (Figure 3), an essential cofactor that facilitates HIV infection (Ma et al., 2004). At the same time, WFDC4 can also play the role by binding to the membrane phospholipid transporter Scramblase 1. During HIV infection of CD4^+^ T cells, Scramblase 1 acts as a viral transporter, delivering HIV into CD4^+^ T cells, and WFDC4 can disrupt this process by binding with Scramblase 1, thereby reducing HIV infection of CD4^+^ T cells (McNeely et al., 1997). Also, elevated expression of WFDC4 is connected with reduced infection of HIV-1 (Pillay et al., 2001).

In the vaginal environment, the overexpressed WFDC14 has been found to be related to the increased resistance to HIV infection (Iqbal et al., 2009). This may be related to the conservative structure of the WFDC domain in WFDC14 and WFDC4’s C-terminal WFDC domain. But the specific mechanisms through which WFDC proteins exert their antiviral effects remain unclear, so further research is needed. For the other WFDC proteins, their antiviral activities still need to be further explored and discovered.

### 2.4 Immunomodulatory function

In addition to their roles in protease inhibition and antimicrobial activity, WFDC proteins are crucial for modulating the immune system. Specifically, WFDC4, WFDC12, and WFDC14 have demonstrated the ability to dampen excessive inflammation by inhibiting key inflammatory mediators and/or pathways (Williams et al., 2006; Glasgow et al., 2015; Lentsch et al., 1999) (Figure 2), such as LPS-induced inflammation. This immunomodulatory function highlights their significance in maintaining immune homeostasis and suggests potential therapeutic implications for conditions characterized by dysregulated inflammation.

Extracellularly, WFDC4 is able to block the formation of LPS-sCD14 complexes, inhibiting the activation of Toll-like receptor (TLR) 4. WFDC4 binds not only to LPS polymers but also to LPS in the LPS-sCD14 complex, potentially reducing LPS-like macrophage metastasis (Ding et al., 1999). Intracellularly, WFDC4 can inhibit the degradation of IκBα, potentially weakening the signaling of TLR2 and TLR4 (Taggart et al., 2002). And more interestingly, WFDC4 can enter the nucleus and bind to DNA. Then WFDC4 competes with NF-κB (p65) for binding to NF-κB DNA sites in gene promoter regions, such as IL-8 and TNF-α, which may be the intracellular mechanism by which WFDC4 blocks LPS and LTA-induced NF-κB expression (Taggart et al., 2005) (Figure 3). Inhibition of p65 binding reduces the transcription of pro-inflammatory cytokines and subsequent reactions, leading to an anti-inflammatory effect. In addition, WFDC4 can upregulate macrophage production of anti-inflammatory/repairing cytokines TGF-β and IL-10 (Sano et al., 2000).

Pre-treatment of THP-1 monocytes with WFDC12 followed by LPS stimulation significantly inhibited interleukin-8 (IL-8) and monocyte chemotactic protein-1 (MCP-1) production (Glasgow et al., 2015). However, further investigation into the mechanism of action is needed. This ability to inhibit cytokines production in LPS-induced monocytes may be useful for managing the inflammatory responses caused by bacterial infection.

WFDC14 also neutralizes LPS extracellularly, similar to WFDC4 (McMichael et al., 2005), and promotes the phagocytosis of apoptotic cells by inhibit human neutrophil elastase (HNE) which can cleave CD14 of macrophages, aiding in the resolution of inflammation (Henriksen et al., 2004). Intracellularly, WFDC14 inhibits LPS-induced JNK phosphorylation and the subsequent phosphorylation of AP-1 subunits ATF2 and c-Jun. Furthermore, WFDC14 can prevent the degradation of NF-κB regulatory proteins—IL-1R-associated kinase 1(IRAK), IκBα, and IκBβ (Figure 3), which is induced by LPS (Butler et al., 2006). Upon activation by LPS, IRAK binds to TRAF6, which is important for the activation of germinal center kinase(GCK) by transiently stabilizing the ubiquitinated GCK peptides. GCK then will activate the JNK, initiating the subsequent signal transduction. However, WFDC14 can interfere with the stability of TRAF6-dependent GCK by influencing the ubiquitin-proteasome mechanism that normally regulates GCK (Butler et al., 2006; Zhong and Kyriakis, 2004). Ultimately, WFDC14 affects the NF-κB and JNK signaling pathways, inhibiting the expression of immune response genes (Figure 3).

## 3 Cancer and WFDC proteins

Cancer remains one of the leading causes of morbidity and mortality worldwide (Sung et al., 2021), presenting unique challenges in diagnosis and treatment. Among the diverse array of cancers, lung, prostate, and ovarian cancers represent significant health concerns due to their prevalence and the complexity of their management. The WFDC protein family, with its distinctive structural and functional features, has emerged as a significant area of research in cancer biology. These proteins, known for their roles in protease inhibition and antimicrobial activities, have also shown potential in the realm of oncology. Understanding how WFDC proteins influence cancer progression, treatment, and prognosis could open new avenues for diagnostic and therapeutic strategies. This section will explore the roles of WFDC proteins in lung, prostate, and ovarian cancers, focusing on their potential as biomarkers and therapeutic targets.

### 3.1 Lung cancer and WFDC2

Currently, cancer-related death is mainly caused by lung cancer in the world, with approximately 2.2 million new cases of lung cancer emerge each year, and about 1.8 million people die from lung cancer (Sung et al., 2021). Despite advances in diagnosis and targeted therapy, the 5-year survival rate for lung cancer patients has not significantly improved. (Siegel et al., 2015). Up to now, there are some non-specific tumor biomarkers such as carcinoembryonic antigen (CEA), neuron-specific enolase (NSE), cytokeratin 19 fragments (CYFRA 21-1), progastrin-releasing peptide (proGRP) and so on, which may promote the early diagnostic rate of lung cancer, but it remains elusive to find the specific markers for the diagnosis of lung cancer (Diamandis et al., 2008). However, it is exactly the late-stage diagnosis of numerous lung cancer patients that leads a greatly reduced survival rate. To improve survival rates, early diagnosis and precise prognostic analysis are essential. Therefore, it is crucial for researchers to find a serological biomarker in patients that can promptly and accurately reflect the development and prognosis of lung cancer. WFDC2 is one of the most promising new markers at present.

Several studies have demonstrated that serum WFDC2 levels are valuable for the diagnosis or prognosis of lung cancer (Nagy et al., 2014; Korkmaz et al., 2018; Liu et al., 2013; Zhong et al., 2017; Lamy et al., 2015; Iwahori et al., 2012; Lan et al., 2016). They found that the levels of serum WFDC2 were generally elevated in lung cancer patients (Table 1), making it easy to distinguish the lung cancer patients from healthy controls (Nagy et al., 2014; Liu et al., 2013; Iwahori et al., 2012). At the same time, if we combined the WFDC2 with carbohydrate antigen 125(CA125) and CEA, the diagnostic effect can be significantly improved (Nagy et al., 2014), which can better help the staging of lung cancer diagnosis. Studies have also highlighted the prognostic value of WFDC2 levels in lung cancer patients. Patients with high serum WFDC2 levels have significantly lower recurrence-free and overall survival compared to those with low levels (Lamy et al., 2015; Iwahori et al., 2012; Lan et al., 2016; Li et al., 2016). This suggests that serum WFDC2 levels are a strong prognostic indicator for lung cancer patients. These studies explored the value of serum WFDC2 levels in non-small cell lung cancer (NSCLC) and demonstrated that WFDC2 is a promising diagnostic and prognostic marker.

In follow-up studies, we should pay more attention to the diagnostic and prognostic value of WFDC proteins in large, randomized samples. At the same time, we can also consider the combination of serum WFDC2 levels with currently used lung cancer markers, which can further improve its accuracy, refining the classification of lung cancer. This is more conducive to the individualized and precise treatment of patients.

### 3.2 Prostate cancer and WFDC1, WFDC2

Prostate cancer (PC) has the highest incidence rate among male malignancies in many regions worldwide. In addition, almost 1.3 million new cases were diagnosed in 2018 (Ferlay et al., 2019). Currently, lots of PCs have favorable survival times if the patients are operated on and/or receive the androgen deprivation therapy. However, once prostate cancer metastasizes from the primary site, the patient’s prognosis becomes worse (Nikhil et al., 2019). Therefore, identifying key substances that cause PC to worsen further and finding early diagnostic biomarkers are crucial. Currently, WFDC protein family members, including WFDC1 and WFDC2, have been studied in relation to prostate cancer (Table 1). Among these, WFDC1 has garnered more research attention.

#### 3.2.1 The role of WFDC1 in prostate cancer

**FIGURE 4:**
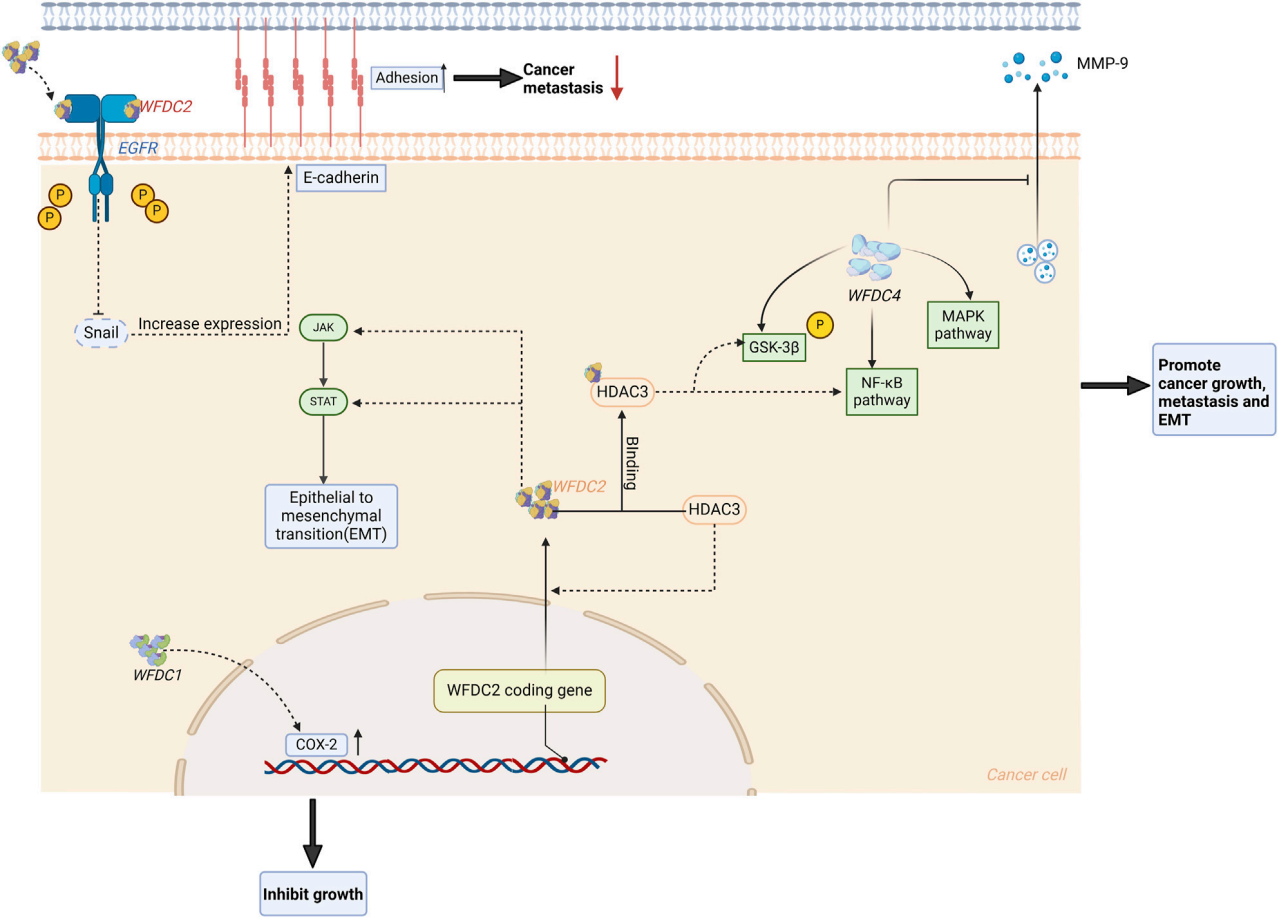
WFDC protein plays a role in the regulation of cancer. For WFDC4, it can increase the expression of PI3K and p-AKT, which subsequently activate the NF-κB and MAPK signaling pathway to promote cancer progression and metastasis. When it comes to WFDC2, it has an effect on the JAK/STAT pathway to increase the EMT. Meanwhile, HDAC3 can induce the expression of WFDC2 and then bind to it to stimulate the GSK-3β and the NF-κB pathway. But extracellularly, WFDC2 can bind to EGFR and inhibit its phosphorylation, which will inhibit the expression of snails. Then this may improve the E-cadherin expression to inhibit the cancer metastasis. HDAC3, histone deacetylase 3. Created with BioRender.com.

One WFDC gene—WFDC1, encodes the prostrate stromal 20 (PS20), a 24 kDa secreted protein. Notably, several studies have reported abnormal expression of WFDC1 in PC patients (Hickman et al., 2016a; Hickman et al., 2012; McAlhany et al., 2003; McAlhany et al., 2004). One of the studies showed that both decreased interstitial WFDC1 expression and increased epithelial expression were associated with higher Gleason scores and reduced survival rates in PC patients (McAlhany et al., 2004). Furthermore, it was also been noted that WFDC1 inhibits the expansion of CD8^+^ T cells and NK cells in the prostate cancer microenvironment and their ability to kill cancer cells (Hickman et al., 2012). A recent study found significantly reduced WFDC1 expression in clinical tumor samples compared to normal prostate tissue, with some tumors exhibiting double WFDC1 deletions (Hickman et al., 2016a). The study reported that WFDC1 affected various cytokine/chemokine pathways, suggesting that WFDC1 exhibited the ability of growth inhibition depending on the COX-2 (Hickman et al., 2016a) (Figure 4). Therefore, we hypothesize that WFDC1 is expressed normally in the prostatic stroma, which induces the expression of COX-2. Moreover, it can regulate the growth-inhibiting and pro-apoptotic environment, thereby inhibiting epithelial growth and thus preventing tumor formation.

The discussion highlights the application value of WFDC1 in prostate cancer, where abnormal expression correlates with poorer prognosis. WFDC1 is a multifunctional which can inhibit CD8^+^ T cells, NK cells, and their ability to kill prostate cancer cells. And in terms of this, further exploration of WFDC1’s role in tumor development is warranted. Additionally, WFDC1’s ability to inhibit epithelial hyperplasia by regulating COX-2 suggests it could be a valuable tumor suppressor.

#### 3.2.2 The role of WFDC2 in prostate cancer

In a recent study of WFDC2 in PC, they reported that WFDC2 was significantly downregulated and had a negative correlation with the Gleason score in PC, which was a surprising result (Xiong et al., 2020). Further studies have shown that the overexpression of WFDC2 has no influence on the proliferation of PC, but can inhibit its metastasis. Up-regulation of WFDC2 resulted in decreased phosphorylation of EGFR, AKT, and GSK-3β, and reduced Snail expression. WFDC2 inhibits EGFR activation by binding to its extracellular domain, thus inhibiting prostate cancer metastasis (Figure 3). At the same time, WFDC2 also inhibits epithelial-mesenchymal transition in prostate cancer through inactivating EGFR signaling. The prognostic value of WFDC2 was also evaluated, revealing that high WFDC2 expression is associated with better survival (Xiong et al., 2020). Currently, a model consisting of PSA, %free PSA, DRE, and WFDC2 has been reported to differentiate clinically significant prostate cancer from non-cancer, potentially reducing unnecessary biopsies (Shore et al., 2023).

This study sheds light on the effect of WFDC2 in the development of PC. WFDC2 exhibits the capability to inhibit the metastasis of PC by blocking the activation of EGFR, suggesting that WFDC2 may be a promising therapeutic target for prostate cancer. And its good prognostic value is also encouraging.

### 3.3 Ovarian cancer and WFDC2, WFDC4

Ovarian cancer constitutes one-third of all gynecological cancers but accounts for 55% of gynecological malignant deaths and 6% of female cancer deaths. Individuals aged between 50 and 70 years old are the people with the highest incidence (Zhang et al., 2021). Both WFDC2 and WFDC4 have been studied in ovarian cancer. WFDC2, in particular, shows great potential for diagnosis and prognosis and has been applied in clinical practice.

#### 3.3.1 The role of WFDC2 in ovarian cancer

WFDC2 is overexpressed in ovarian cancer, particularly in endometrioid ovarian cancer (Drapkin et al., 2005), and interestingly, a current study reported WFDC2 and resectability in advanced epithelial ovarian cancer patients (Muhammad et al., 2023). Initial WFDC2 level can be an independent factor influencing the prognosis of patients with stage III serous ovarian cancer (Yang et al., 2023). Moreover, the overexpression of WFDC2 is also associated with the proliferation, metastasis, and invasion of ovarian cancer (Gao et al., 2011), attributed to the change of several signaling pathways (Figure 4). One study reported that WFDC2 protein stimulates SKOV-3 cell proliferation via activation of the PI3K/AKT signaling pathway (James et al., 2018), with the highest viability observed at 0.2 μg/mL WFDC2 protein (Wang et al., 2015). At the same time, they also studied the effect of WFDC2 protein on the carboplatin-induced apoptosis, revealing that WFDC2 protein can weaken the drug’s ability to induce apoptosis. Besides, histone deacetylase 3 (HDAC3) is overexpressed in ovarian cancer, inducing the WFDC2 expression and binding to it (Lou et al., 2019). Then this complex will also activate the PI3K/AKT pathway to affect the GSK-3β and NF-κB pathway, which stimulates proliferation. Meanwhile, the JAK/STAT3 pathway can be inhibited by slicing WFDC2 which will suppress the cancer growth (Wang et al., 2019) (Figure 4).

WFDC2 has also been extensively studied for ovarian cancer diagnosis. Researchers compared transvaginal ultrasound (TVU) with serum WFDC2 levels for ovarian cancer screening, finding that serum WFDC2 is more effective (Urban et al., 2011). This approach effectively reduces unnecessary surgeries and makes screening more convenient. Additionally, combining CA125 with WFDC2 is more specific for diagnosing ovarian cancer than using CA125 alone (Dochez et al., 2019). A 4-marker panel consisting of CA125, WFDC2, E-cadherin(E-CAD), and IL-6 was also studied, showing the highest accuracy, both alone and in combination with WFDC2 (Han et al., 2018).

#### 3.3.2 The role of WFDC4 in ovarian cancer

WFDC4 is also overexpressed in ovarian cancer (Table 1), and is closely related to the progression of ovarian cancer (Hough et al., 2001). Studies have reported that exogenous WFDC4 protein significantly increases the ability of metastasis and invasion in ovarian tumor cells. Additional research found that WFDC4 treatment significantly increased PI3K, p-AKT (S473), and p-AKT (T308) levels in ovarian cancer cells (Figure 4). This suggests that WFDC4 secreted by cancer-associated fibroblasts (CAFs) substantially affects the PI3K-AKT signaling pathway, which is linked to tumor growth (Sun et al., 2022). It has also been reported that WFDC4 may stimulate the invasion of ovarian cancer, partly through its antiprotease activity to reduce the releasing of MMP-9, while its role in inducing MMP-9 does not depend on protease inhibitory activity (Hoskins et al., 2011).

From the above studies, we can find that WFDC2 and WFDC4 are crucial for the development and progression of ovarian cancer. Therefore, WFDC4, along with WFDC2—which affects multiple cancer-associated signaling pathways—could be considered as potential therapeutic targets. The diagnostic value of the WFDC2 group is well established and has been implemented in clinical practice.

## 4 Inflammatory disease and WFDC proteins

Inflammation is a general reaction of the organism to local tissue changes of various origins. According to the principles of general pathology, inflammation is a complex pathological process, both local and systemic, that underlies the pathogenesis of many diseases and presents with diverse localizations and symptoms (Bennett et al., 2018). Current treatments include corticosteroids and nonsteroidal anti-inflammatory drugs (NSAIDs), which can cause systemic toxicity, as well as biologics, which may increase the risk of infection. Given the diverse functions of WFDC proteins, including antiprotease, antimicrobial, and immunomodulatory functions, we may consider that WFDC proteins can play a significant role in various inflammatory diseases, such as the recently reported value of WFDC proteins in interstitial lung disease, psoriasis, and atopic dermatitis.

### 4.1 Interstitial lung disease and WFDC2

The typical manifestation of interstitial lung disease (ILD) is the inflammation and/or fibrosis of lung tissue, and it is a major complication of some systemic inflammatory diseases, including rheumatoid arthritis (RA), systemic sclerosis (SSc, scleroderma), idiopathic inflammatory myopathy (IIM) (Zhang et al., 2020; Liang et al., 2021; Lin et al., 2022; Sun et al., 2023). ILD is a common and severe complication of these systemic inflammatory diseases and is a significant cause of death. Therefore, it is extremely important to identify a reliable predictor of the risk of ILD in these patients and an effective method for assessing the disease severity.

Approximately 60% of RA patients are also diagnosed with ILD, and both two studies reported the increased levels of serum WFDC2 in RA-ILD patients when compared to the RA-non-ILD patients and healthy controls (Liang et al., 2021; Lin et al., 2022). Another study found that ILD prevalence was higher in the WFDC2-positive group compared to the negative group (Liang et al., 2021). Also, the level of serum WFDC2 is positively related to the extent of lung damage, indicating the severity of the disease. In patients with SSc, high-resolution computed tomography (HRCT) revealed that ILD was involved in about two-thirds of patients, and one study also reported elevated WFDC2 levels in serum and BALF from SSc-ILD patients compared to those without ILD. Additionally, a semi-quantitative measure of ILD on CT scans was positively correlated with WFDC2 levels (Zhang et al., 2020). Similar results were observed in patients with IIM (Sun et al., 2023).

The above is the current study of WFDC2 (WFDC2) in interstitial lung disease, but from the above reports, we can still understand the potential application value of serum WFDC2 in the prediction and diagnosis of ILD. In particular, contrasted to the current main diagnostic test—CT scan, serum WFDC2 detection is simpler and faster, potentially aiding in treatment and further diagnostic evaluations. However, in order to further validate the accuracy of serum WFDC2 levels, further larger-scale studies are needed.

### 4.2 Psoriasis and WFDC12, WFDC4, WFDC14

Psoriasis is a kind of autoimmune inflammatory disease that is specific of the skin, and it is characterized by extreme thickened skin, chronic inflammation of the dermis, erythema, and skin scales. The expression of WFDC12 was found to be induced during late differentiation of keratinocytes while this WFDC protein also helped regulate the activity of proteases in the stratum corneum (Kalinina et al., 2021). Among the WFDC family, the high expression of WFDC4, WFDC12, and WFDC14 have been noted in the lesions of psoriasis.

#### 4.2.1 The role of WFDC12 in psoriasis

In psoriasis patients, WFDC12 expression increases in skin lesions and correlates with the severity of pathological features (Kalinina et al., 2020; Zhao et al., 2022) (Table 1). WFDC12 is considered to have a potential effect on the pathogenesis of psoriasis. According to a study, WFDC12 was observed to be vastly expressed in the keratinocytes (KCs) of K14-WFDC12 transgenic mice. Following Imiquimod (IMQ) induction, increased infiltration of lesion areas and lymph node dendritic cells was observed, along with elevated IL-12 release in the lesion areas (Zhao et al., 2022). Similarly, WFDC12 overexpression also increased the expression of the CCL19 chemokine in skin lesions, thereby activating dendritic cells. It can be hypothesized that the infiltration of Langerhans cells (LCs) and monocyte-derived dendritic cells (moDDCs) may be interfered with by WFDC12 in the mice of IMQ-induced psoriasis, potentially regulating the immune function. The experiments conducted suggested that K14-WFDC12 mice had significantly more T-helper 1 (Th1) cells infiltrated lymph nodes compared to WT mice, with elevated IFN-γ levels (Zhao et al., 2022). Clinical studies have shown that the number of Th1 cells in psoriasis patients increases significantly, indicating that Th1 cells have an essential effect on the pathogenesis of psoriasis. With the impact of chemokines secreted by KC, the Th1 cells will move to the skin lesions in lymph nodes, secreting key inflammatory cytokines IFN-γ and the other related cytokines, aggravating its symptoms (Quaglino et al., 2011).

Therefore, WFDC12 likely facilitates the infiltration of LCs into the epidermis and lymph nodes, and this elevated production of IL-12 then helps in promoting the differentiation of lymph nodes and peripheral blood Th1 cells, resulting in the secretion of IFN-γ in the damaged region of the IMQ-induced psoriasis mice. Further analysis revealed downregulation of RDH10 and DHRS9 expression in transgenic mice lesions, indicating blocked tretinoin ATRA production and reduced tretinoin ATRA synthesis in the IMQ-induced psoriasis model (Zhao et al., 2022). Besides, the reduction of tretinoin did inhibit the development of LC and moDDCs and also inhibited the differentiation of T cells by impairing the DCs, suggesting that the differentiation of DCs and Th1 cells may be activated by the lower tretinoin concentration by interfering with the RAR-RXR transcription-associated genes.

#### 4.2.2 The role of WFDC4 and WFDC14 in psoriasis

In the skin lesions of psoriasis patients, WFDC4 and WFDC14, two extensively studied WFDC proteins, are overexpressed. WFDC4 affects the pathogenic process of psoriasis by limiting the formation of neutrophil extracellular traps (Zabieglo et al., 2015), meanwhile, by activating the extracellular DNA to enhance the secretion of IFN-α from pDCs (Skrzeczynska-Moncznik et al., 2012). However, serum levels of WFDC14 are clinically associated with the pathogenic extent of psoriasis and the inflammatory markers (Meyer-Hoffert et al., 2004).

Based on the discussion above, it is evident that WFDC4, WFDC12, and WFDC14 are all highly expressed in psoriasis lesions, with WFDC12 notably impacting psoriasis development through multiple mechanisms. However, the roles of WFDC4 and WFDC14 in psoriasis are not as well understood and warrant further investigation. WFDC12, due to its significant involvement in the progression of psoriasis, holds potential as a target for targeted therapies. Nevertheless, the practicality and effectiveness of such approaches require thorough evaluation. Further research is needed to better understand the precise mechanisms of these WFDC proteins and to assess the potential for WFDC12 as a therapeutic target in psoriasis.

### 4.3 Atopic dermatitis and WFDC12

Atopic dermatitis (AD) is a recurrent, chronic, non-infectious inflammatory skin disease characterized by persistent itching of the skin. It occurs mainly in children, with a prevalence of up to 20% (Arrais et al., 2019; Asher et al., 2006). The pathogenesis of AD involves a variety of complex factors such as genetic, environmental and psychological factors. And the existing pharmacological treatments have limited efficacy and certain side effects. Therefore, there is an urgent need to further investigate the pathogenesis of AD and related functional molecules, in order to lay the foundation for the development of new drug targets and clinical treatments. WFDC12 is a key important factors, which is highly expressed in the skin lesions of AD patients (Kalinina et al., 2021).

It is well known that inflammation is an important part in the pathogenesis of AD. A recent study identified a link between overexpressed WFDC12 in skin lesions of DNFB-induced transgenic mice and AD development (Li et al., 2023). They found that overexpression of WFDC12 in AD lesions was associated with clinical features of AD and might be involved in the development of AD. WFDC12 might be involved in the development of AD in three ways: 1) Overexpression of specific WFDC12 in the epidermis promotes the migration of mAPCs from the skin to the lymph nodes to accelerate the differentiation of Th cells and enhancing the epidermal immune-inflammatory response; 2) Keratinocyte-specific overexpression of WFDC12 may upregulate the expression of ALOX12 and ALOX15, activating the lipoxygenase ALOX12/15 pathway in the epidermal arachidonic acid(AA) metabolism pathway, and promotes the accumulation of the inflammatory mediators of AA metabolite 12-HETE and 15-HETE. This leads to increased inflammatory mediators 12-HETE and 15-HETE, exacerbating the DNFB-induced inflammatory response and AD symptoms in mice; 3) WFDC12 may also promote platelet-activating factor(PAF) accumulation and activate AA metabolism by inhibiting serine protease activities, including platelet-activating factor acetylhydrolase and Cytochrome P450 protein 4F14. This promotes the accumulation of PAF and activates AA metabolism in the lipoxygenase pathway, enhancing the production of inflammatory lipid mediators involved in the pathogenesis of AD (Li et al., 2023).

It is evident that overexpression of WFDC12 can significantly influence the progression of AD through multiple pathways. One key mechanism is its promotion of the ALOX12/15 metabolic pathway, which plays a crucial role in lipid metabolism and inflammation. Additionally, WFDC12 overexpression is associated with increased accumulation of PAF, a lipid mediator linked to inflammation and cellular damage. These interactions suggest that WFDC12 may be a promising therapeutic target for AD, which will point out the direction for the development of related drugs and clinical treatment.

## 5 Preeclampsia and WFDC1

Preeclampsia is a severe medical condition characterized by hypertension during pregnancy, which poses significant risks to both maternal and fetal health. It is crucial to achieve accurate diagnosis and prognosis due to the potential complications associated with the condition, such as seizures (eclampsia), organ damage, and cardiovascular complications. Therefore, ensuring early and accurate assessment of preeclampsia is essential for optimizing both maternal and fetal outcomes during pregnancy. A recent study on preeclampsia has elucidated a potential association between the WFDC1 and preeclamsia, which could aid in the diagnosis and/or prognosis of preeclampsia (Rajakumar et al., 2011).

Researchers have performed whole transcriptome analyses for healthy pregnant women and those with preeclampsia (Rajakumar et al., 2011). They observed that the expression of several genes, including FN1, MMP9, WFDC1, BIRC5, CAV1, GATA1, and E2F1, differed between healthy pregnant women and those with preeclampsia. WFDC1 is exactly what we’re going to discuss. Monoclonal antibodies were used to confirm differences in protein levels of selected gene products. The study demonstrated that these proteins were upregulated in PBMCs of women with preeclampsia (Rajakumar et al., 2011). In patients with preeclampsia, WFDC1 may be valuable, which can provide the value of diagnosis and prognosis, according to the elevated expression of WFDC1 at the protein level.

However, there are several limitations at present. For instance, studies on WFDC1 in preeclampsia are limited, and existing research only measures differences in WFDC1 protein expression between patients and healthy controls, without evaluating its diagnostic or prognostic value. Meanwhile, preeclampsia is the main obstetric complication that can lead to fetal and maternal morbidity or mortality, highlighting the need for a detailed study of its pathogenesis. In this study, WFDC1 was identified as a key gene presenting differential expression between patients and healthy controls. Therefore, follow-up studies may further explore the role of the different gene expressions in the pathogenesis of the disease, or determine the prognostic value of the expression of WFDC1 in preeclampsia.

## 6 Heart failure and WFDC2

For most cardiovascular diseases, which consist of myocardial infarction, hypertension, valvular disease, cardiomyopathy, and so on, heart failure (HF) is the typical syndrome (Stewart et al., 2001; McMurray et al., 2012). HF, which refers to a complicated syndrome, is described by decreased cardiac function, resulting in inadequate cardiac output to satisfy the metabolic demands of peripheral tissues. To date, in clinical practice, several specific clinical markers of the heart, such as natriuretic peptides (ANP and BNP) and high-sensitivity troponin, which are extensively applied (McMurray et al., 2012). Over the past decades, numerous studies have been conducted to determine new biomarkers for HF which can provide better or supplementary diagnostic modalities and aid in prognosis and disease stratification.

Several studies have reported the application of WFDC2 in the diagnosis of HF, including acute heart failure in people with chronic kidney disease (Piek et al., 2017; Castillo Perez et al., 2022; Huang et al., 2020; Piek et al., 2018; de Boer et al., 2013; Dong et al., 2023). These researchers reported that significantly higher levels of WFDC2 were observed in patients with HF when compared to the healthy controls (Table 1), and increased with the NYHA grades. Additionally, patient WFDC2 levels correlated with several risk factors for heart failure, including sex, age, smoking, diabetes, and the B-type natriuretic peptide N-terminal precursor hormone (NT-proBNP). According to a multivariate model, NT-proBNP, galactolectin-3, high-sensitivity troponin T, and smoking were identified as the related factors to WFDC2 (Piek et al., 2017). The study also includes other HF biomarkers under investigation. Addition of WFDC2 to clinical evaluations increased the area under the ROC curve, indicating improved diagnostic performance. Additionally, after adding WFDC2 to the clinical model, the composite discrimination and net reclassification index were promoted significantly (de Boer et al., 2013). Interestingly, there was also a single-center retrospective study that analyzed the relationship between new-onset HF and serum WFDC2 after acute coronary syndromes in women. It found that serum WFDC2 levels were significantly higher in patients with adverse events compared to those without. (Yan et al., 2023).

HF is a multifaceted and heterogeneous disease where precise diagnosis and careful grading are crucial for guiding treatment and predicting outcomes. And most of the above studies use WFDC2 in combination with existing HF biomarkers, which greatly increases the accuracy of HF diagnosis and prognosis. By leveraging WFDC2 in conjunction with traditional markers, clinicians can better stratify patients, tailor therapies more effectively, and anticipate clinical outcomes more accurately in HF management strategies. Further research is warranted to fully elucidate the role of WFDC2 and its potential as a biomarker in the comprehensive management of heart failure.

## 7 Male contraception and WFDC7

WFDC7 (Eppin) is a mammalian conserved sperm-binding protein with a WFDC domain at its N-terminal and a Kunitz protease inhibitor domain at its C-terminal (Figure 1). Previous studies have shown that spermatozoa are coated with WFDC7 on both the head and tail (Wang et al., 2005), and WFDC7 is a component of the ejaculate coagulation. Upon entering the ejaculatory tract, the WFDC7 coat on the sperm becomes bound and saturated by semenogelin (SG), the primary protein component of semen. This forms a protective substance for the sperm (De Lamirande et al., 2001). After ejaculation, PSA hydrolyzes the coagulum, thereby releasing spermatozoa motility.

Now, a study has reported that immunization of adult male rhesus monkeys with anti-WFDC7 antibodies results in reduced semen coagulation and decreased fertility (O’Rand et al., 2006). The researchers hypothesize that the WFDC7 antibodies bind to WFDC7, causing the blocking of SG’s binding site. This results in two effects: first, semen fails to coagulate; and second, WFDC7 antibodies bind to WFDC7, producing an effect similar to the action of sperm motility inhibitory peptide, which impairs sperm motility (O’Rand et al., 2006). Recent studies evaluated the effects of antibodies targeting specific WFDC7 epitopes (Q20E antibody for Gln 20-Glu 39, S21C and F21C antibodies for Ser 103-Cys 123 and Phe 90-C110) on sperm motility and fertilization capacity in mice (Silva et al., 2021). Computer-assisted sperm analysis showed that co-incubation with the S21C antibody (but not the F21C antibody) reduced progressive and overactivated motility and impaired kinematic parameters, such as linear speed, average path velocity, and straightness, as well as curved speed, lateral head movement amplitude, and linearity. In contrast, the Q20E antibody had a milder inhibitory effect on forward motility and kinematic parameters. Unfortunately, similar observations *in vivo* are still needed for confirmation. And according to an updated report, WFDC7 has a binding pocket accommodating SEMG1^Glu229-Gln247^, EP055, and EP012, which may serve as the target of the new contraceptive drugs (Gomes et al., 2023).

Currently, two widely used methods of male contraception are available, namely condom use and vasectomy. However, both methods have drawbacks - condom use may reduce sensation, while vasectomy requires surgical ligation and may cause harm to the body. Hormonal drugs are another option for contraception, but this method has a short duration of action and long-term use has significant side effects on the human body. Therefore, this research offers a new perspective. The interaction between WFDC7 and SG is a key process of sperm motility. Designing antibodies against WFDC7 to inhibit its interaction with SG could provide a new contraceptive method. This way of vaccination not only minimize harm to the human body, but also has a long efficacy period, with multiple injections will not have adverse effects on the human body. However, the feasibility of this method as well as the result *in vivo* experiments remains to be confirmed. This finding implies that WFDC7 could provide a prospective target for male contraception.

## 8 The current status of clinical research

The exploration of WFDC proteins in clinical research is an emerging and promising area, particularly focusing on WFDC4 and WFDC14. WFDC4, also known as secretory leukocyte protease inhibitor (SLPI), has garnered attention for its potential therapeutic benefits in inflammatory lung diseases, with ongoing studies examining its efficacy in reducing inflammatory markers and improving drug delivery methods. Meanwhile, WFDC14 is being investigated for its therapeutic applications in various conditions, including esophageal cancer and inflammatory bowel disease, with early trials showing encouraging results. This section will delve into the current state of clinical research on these proteins, highlighting recent findings, advancements in drug delivery technologies, and the potential for broader applications in treating inflammatory conditions and enhancing clinical outcomes.

First, we will discuss the therapeutic application of WFDC4. It is known that WFDC4 is the major antiproteases in upper respiratory tract epithelial cells under physiological conditions. While data from some patients with chronic pathology suggest that WFDC4 is usually inactivated in inflammatory secretions, either by the action of the host or microbial products (Sallenave, 2010), this justifies attempts to supplement with antiproteases in clinical protocols. Studies have reported the efficacy of recombinant WFDC4 (rWFDC4) nebulizers in patients with inflammatory lung disease. Nebulization of rWFDC4 significantly reduced active NE levels and caused a noticeable decrease in IL-8 levels and neutrophil counts in CF epithelial lining fluid (ELF) (McElvaney et al., 1992). However, in normal volunteers, the deposition of rWFDC4 is uniform in all lobes, while in patients with CF or emphysema, aerosol deposition is shown only in well-ventilated areas (Stolk et al., 1995). This result suggests that the application of rWFDC4 by aerosol therapy can protect the normal lung tissue from further proteolytic injury, while the severely inflamed areas or poorly ventilated bulla may benefit less.

A pharmacokinetic study on rWFDC4 showed that 1 h after inhalation of rWFDC4 (100 mg single time) in normal people, the level of rWFDC4 and the ability of anti-NE increased significantly in airway ELF, and gradually decreased for 4∼12 h (McElvaney et al., 1993). Interestingly, there was no difference in WFDC4 levels or anti-NE activity between the group treated continuously for a week and the group given a single rWFDC4 dose, indicating that rWFDC4 does not accumulate on the respiratory epithelium. conversely, there was no decrease in active NE levels in the ELF of volunteers treated with 50 mg twice daily, which may indicate that this dosage regimen did not sufficiently increase ELF WFDC4 levels (McElvaney et al., 1993).

However, effective drug delivery to inflammatory lungs is challenging. Studies have attempted to encapsulate inhibitors in liposomes to prevent unwanted proteolysis, and recent research shows that encapsulated WFDC4 is protected from cathepsin L degradation (Gibbons et al., 2010). It is preferred to apply the liposome-encapsulated drugs in the aerosolization for its several advantages, such as constant release, improved biocompatibility, and better loading capacity. Recently, a study attempted to encapsulate proteins within a nanoparticle composed of protein for the first time (Tarhini et al., 2020). They prepared human serum albumin (HSA) nanoparticles and encapsulated WFDC4 within them. Moreover, the results showed that WFDC4 encapsulation in HSA nanoparticles did not impair its inhibitory ability against the bacteria and NE. This may provide new ideas for us to better target the delivery of drugs.

At the same time, we know that WFDC4 can exert anti-inflammatory effects by modulating intracellular signaling pathways. Therefore, identifying the techniques that can enhance the expression of genes is encouraging. Elevated Nrf2 activity has been reported to associated with the elevated expression of WFDC4. Additionally, in cruciferous vegetables, researchers determined a kind of isothiocyanate—sulforaphane, which enhances the activity of Nrf2. After 48 h of consuming broccoli homogenate containing abundant sulforaphane, there was a noticeable elevation of the WFDC4 concentrations in the nasal lavage solution of volunteers (Meyer et al., 2013). This approach may play a role in preventive measures in patients with chronic airway disease by increasing WFDC4 levels.

As for WFDC14, a phase II clinical study evaluated the effect after perioperative intravenous bolus of 200 mg WFDC14 in patients undergoing esophageal cancer resection. The need for postoperative intensive care was significantly reduced, with 63% discharged after 1 day (Henriksen, 2014). At the same time, the therapeutic application of WFDC14 has demonstrated its effectiveness in many animal experiments. Non-pathogenic probiotic lactic acid bacteria (LAB) have been engineered, which can be applied to produce and convey the WFDC14 in murine models (Motta et al., 2012). In addition, the immunostaining of WFDC14 suggested that a high level of it was detected within the mucosa, distant from the location of bacteria, supporting its feasibility. After being treated with these bacteria, a 60% reduction in the activity of the elastin proteolysis (Motta et al., 2012). Despite challenges in regulating WFDC14 expression by LAB, this proof-of-principle study paves the way for clinical application in inflammatory bowel disease. Additionally, several studies have also investigated the efficacy of WFDC14 in animal models for ischemia-reperfusion injury, including ischemia-reperfusion injury of skeletal muscle and myocardium, and WFDC14 has shown a certain therapeutic effect and protective effect (Henriksen, 2014; Crinnion et al., 1994; Tiefenbacher et al., 1997).

Although there are some clinical studies on WFDC proteins, many studies are limited to WFDC4 and WFDC14. Most of the research on WFDC4 primarily involves the use of WFDC4 nebulizers in lung inflammatory diseases. One difficulty here is to target the delivery of drugs to areas of inflammation in the lungs. Excess mucus in the airways of patients not only interferes the sedimentation of aerosols, but also serves as a barrier to drugs spreading. Then how to deliver drugs more efficiently and accurately will be the place where breakthroughs are needed in the future, and drugs that can dilute mucus and ensure that the active proteases in the mucus are not released. Currently, the application of these WFDC proteins focuses on their antiprotease and immunomodulatory functions, with limited exploration of their antibacterial and antiviral activities. Future research should aim to develop new antibacterial and antiviral agents. More clinical studies should also be conducted on other WFDC proteins, such as WFDC12 and WFDC1, exploring their functions, including antiprotease, antibacterial, and tumor suppression.

## 9 Discussion

The proteins of WFDC family exhibit both notable similarities and distinct differences in their structure and functions. Structurally, all WFDC family members share a common WFDC domain with a conserved four-disulfide core motif. This structural feature is pivotal for their stability and is integral to their functional roles across various biological contexts (Eisenberg et al., 1990). Functionally, WFDC proteins commonly exhibit protease inhibition activity, which plays a crucial role in regulating inflammatory responses and immune functions (Moreau et al., 2008; Williams et al., 2006; Lentsch et al., 1999). Some members also display antimicrobial properties, contributing to pathogen defense (Baranger et al., 2008; Hiemstra et al., 1996).

Despite these similarities, there are significant differences in function and expression among WFDC family members. For instance, WFDC1 is implicated in prostate cancer, where it modulates the COX-2 pathway and influences immune cell activities, potentially serving as a tumor suppressor (Hickman et al., 2016a). On the other hand, WFDC2 has been identified as a prominent biomarker in various cancers, including lung, ovarian, and prostate cancers, with roles in tumor metastasis and prognosis due to its interaction with EGFR and its ability to modulate cancer cell migration (Xiong et al., 2020; Nagy et al., 2014; James et al., 2018). WFDC4, predominantly studied in ovarian cancer, affects tumor invasion and metastasis by regulating the PI3K-AKT signaling pathway and MMP-9 secretion (Sun et al., 2022; Hoskins et al., 2011). Furthermore, the expression patterns of these proteins further underscore their distinct roles. WFDC2 is commonly upregulated in multiple cancer types and inflammatory diseases and WFDC12 is upregulated in some inflammatory diseases, suggesting their utility as a diagnostic and prognostic marker. Conversely, WFDC1’s expression is often reduced in prostate cancer stroma, suggesting its potential role in cancer suppression.

Besides the WFDC domain, the transglutaminase substrate domain in certain WFDC proteins, including WFDC4, WFDC14, WFDC12, WFDC1, and trappin-2 (the precursor of WFDC14), is crucial for their functions. (Glasgow et al., 2015; Nara et al., 1994; Baranger et al., 2011; Hickman et al., 2016b). All these proteins can cross-link to the ECM by interacting with transglutaminase 2 (TG2) (Muto et al., 2007). However, the consensus sequence GQDPVK, a recognized transglutaminase substrate, is reported to be present only in WFDC14 and trappin-2. In contrast to trappin-2, SLPI lacks a motif containing the consensus sequence GQDPVK, however, the N-terminal domain of SLPI contains a similar motif having the W_30_QCPGK_35_ sequence. Mass spectrometry reveals that most reactive lysine and glutamine residues in SLPI are located in its N-terminal elastin-like domain, while in trappin-2, these residues are found in the N-terminal cementoin domain and elastin portion (Baranger et al., 2011). Although WFDC12 and WFDC1 have been reported to cross-link to the ECM via TG2 (Glasgow et al., 2015; Hickman et al., 2016b), detailed analyses of their specific action sites are lacking. This transglutaminase substrate domain mediates the cross-linking of the corresponding WFDC proteins to the ECM components fibronectin and elastin, thereby anchoring them to the ECM and thus carrying out the corresponding protease inhibitor and immunomodulatory functions.

WFDC proteins have been found to be upregulated in many diseases, such as WFDC1, WFDC2, WFDC12, WFDC14 are upregulated in cancers, inflammatory diseases and so on. However, the mechanisms of gene regulation for these proteins need further investigation. Additionally, WFDC proteins are processed after synthesis by various proteases: 1) WFDC14 is cleaved from its precursor trappin-2 by the cathepsin -L and -K^18^; 2) WFDC4 is inactivated by cleavage by the cathepsin-B, L, and S (Taggart et al., 2001); 3) Cathepsin-L cleaves WFDC1 at the C-terminal end to produce a WFDC1 variant with a lower molecular weight (Hickman et al., 2016b).

Based on the above discussions, WFDC4 is the most ubiquitously employed protein in the WFDC protein family. WFDC4 is noteworthy for its extensive range of functions, including antiprotease, antibacterial and anti-inflammatory activities. Moreover, it has been involved in the development of various diseases, such as ovarian cancer and psoriasis. WFDC2, also known as HE4, exhibits considerable promise as a biomarker and is highly valued for its diagnostic and prognostic potential in various illnesses. The remaining WFDC proteins will not be delineated here.

However, most of the studies have a limitation, that is, the sample size of the included studies is not large, which makes the potential of WFDC proteins as biomarkers still not accurate enough. Subsequent studies on the WFDC family of proteins should validate their diagnostic and prognostic value across a border range of conditions. Additionally, we should consider that since WFDC2 has diagnostic or prognostic value in many diseases, this brings a difficult point, that is, the specificity of WFDC2 as a biomarker will be reduced. Then, we should consider combining WFDC2 with some pre-existing specific markers of these diseases, which may further enhance diagnostic or prognostic capabilities, and this has proven to be effective.

Certain WFDC proteins play a role in the pathogenesis of various diseases, suggesting their potential as drug targets for therapeutic intervention. Nonetheless, it is necessary to confirm the practicality and feasibility of this approach through additional clinical studies. To date, researches on the WFDC family have predominantly focused on WFDC4 and WFDC14, although several studies have explored WFDC2’s potential as a biomarker, without examining the specific molecular background and associated mechanisms. Applications such as drug development of WFDC protein are currently limited to WFDC4 and WFDC14, and the scope of application is also relatively limited. If follow-up research can expand the research scope of WFDC protein and deeply explore its related mechanism, it will greatly promote their application in diseases.

In conclusion, the WFDC protein family, particularly WFDC4 and WFDC2, exhibits multifaceted roles in health and disease, from their antimicrobial and anti-inflammatory functions to their potential as biomarkers in various pathologies. Despite promising advancements, the field faces challenges including sample size limitations in studies and the need for deeper molecular understanding. Future research should focus on expanding the scope of investigation to include less studied WFDC proteins and elucidating their specific roles and mechanisms. This approach will help us harness the full therapeutic and diagnostic potential of WFDC proteins, paving the way for novel drug targets and improved clinical outcomes across diverse medical contexts.
